# Bone marrow-derived cells and their conditioned medium induce microvascular repair in uremic rats by stimulation of endogenous repair mechanisms

**DOI:** 10.1038/s41598-017-09883-x

**Published:** 2017-08-25

**Authors:** Lina Golle, Hans U. Gerth, Katrin Beul, Barbara Heitplatz, Peter Barth, Manfred Fobker, Hermann Pavenstädt, Giovana S. Di Marco, Marcus Brand

**Affiliations:** 1 Department of Internal Medicine D, University Hospital Muenster, Muenster, Germany; 2 Department of Pathology, University Hospital Muenster, Muenster, Germany; 3 Centre for Laboratory Medicine, University of Münster, Münster, Germany

## Abstract

The reduced number of circulating stem/progenitor cells that is found in chronic kidney disease (CKD) patients may contribute to impaired angiogenic repair and decreased capillary density in the heart. Cell therapy with bone marrow-derived cells (BMDCs) has been shown to induce positive effects on the microvasculature and cardiac function, most likely due to secretion of growth factors and cytokines, all of which are present in the conditioned medium (CM); however, this is controversial. Here we showed that treatment with BMDC or CM restored vascular density and decreased the extent of fibrosis in a rat model of CKD, the 5/6 nephrectomy. Engraftment and differentiation of exogenous BMDCs could not be detected. Yet CM led to the mobilization and infiltration of endogenous circulating cells into the heart. Cell recruitment was facilitated by the local expression of pro-inflammatory factors such as the macrophage chemoattractant protein-1, interleukin-6, and endothelial adhesion molecules. Consistently, *in vitro* assays showed that CM increased endothelial adhesiveness to circulating cells by upregulating the expression of adhesion molecules, and stimulated angiogenesis/endothelial tube formation. Overall, our results suggest that both treatments exert vasculoprotective effects on the heart of uremic rats by stimulating endogenous repair mechanisms.

## Introduction

Chronic kidney disease (CKD) is closely associated with cardiovascular disease and a high risk of death^1, 2^. The majority of patients with CKD die prematurely due to cardiovascular comorbidities, even before beginning dialysis. Microvascular remodeling has been observed throughout the myocardium of patients with CKD and that of uremic animals^3, 4^. Impaired angiogenesis participated critically in ventricular remodeling, heart dysfunction, and subsequent heart failure^4, 5^. Diminished capillary density is not restricted to the heart, but it has been observed in the skin of dialysis patients, as well as the kidneys and hind limbs of animals with induced CKD^4, 6–8^. Thus, CKD can be considered a state of anti-angiogenesis due to the accumulation of factors that negatively affect endothelial function^9^. Several perturbations that are present in renal failure may play a role, such as a decreased number and impairment of circulating stem/progenitor cells, which participate in the process of tissue repair^3, 7, 10^.

Bone marrow-derived cells (BMDCs) are a pool of pluripotent stem and progenitor cells that include, among others, hematopoietic stem cells, mesenchymal stromal cells, and endothelial progenitor cells^11, 12^, which secrete a variety of growth factors, cytokines, exosomes, and microvesicles^13, 14^. Various clinical trials have shown that cardiac function improved in patients with acute myocardial infarction who underwent BMDC therapy^15, 16^. The therapy’s positive effect on the microvasculature was also observed in experimental studies that showed increased capillary density in an ischemic hind limb model after BMDC administration. However, engraftment of these cells into the ischemic area and differentiation into cardiac cells or endothelial cells appear to be minimal or even absent^17, 18^. These findings emphasize the endocrine mechanism of stem cell repair rather than engraftment itself. Conversely, BMDC-conditioned medium (CM) can potentially induce angiogenesis and reduce glomerular injury to the kidney in patients with CKD^19^, but it also displays long-lasting therapeutic effects in other diseases such as spinal cord injury or uveitis^11, 20^.

Stimulation of angiogenesis in the ischemic heart is an important step in cardiac repair. In adults, angiogenesis is regulated not only by different growth factors^21, 22^ but also by the recruitment of marrow-derived endothelial as well as hematopoietic cells (collectively defined here as endogenous BMDCs)^23, 24^. Once they infiltrate the target tissue, these cells function in a paracrine fashion to regulate a complex process that involves inflammation, angiogenesis, and tissue repair^25–27^.

Considering that (1) CKD is associated with a decreased number of circulating progenitor cells, (2) this reduction represents a higher risk of future cardiovascular events and cardiovascular death as observed in a meta-analysis^28^, and (3) these cells (and their CM) are able to promote angiogenesis and vascular repair, it is reasonable to propose therapy with BMDCs as an alternative to replenish the stem and progenitor pool in CKD, or mimic their endocrine mode of action using therapy with the CM.

Here we provide evidence that treatment with exogenous BMDCs or CM exerts vasculoprotective effects on the heart of uremic rats by stimulating the endogenous vasculogenic potential; i.e., through the mobilization of endogenous BMDCs and vasculogenic progenitors in the circulation, cell infiltration into the heart, and up-regulation of factors that positively regulate angiogenesis.

## Results

**Figure 1:**
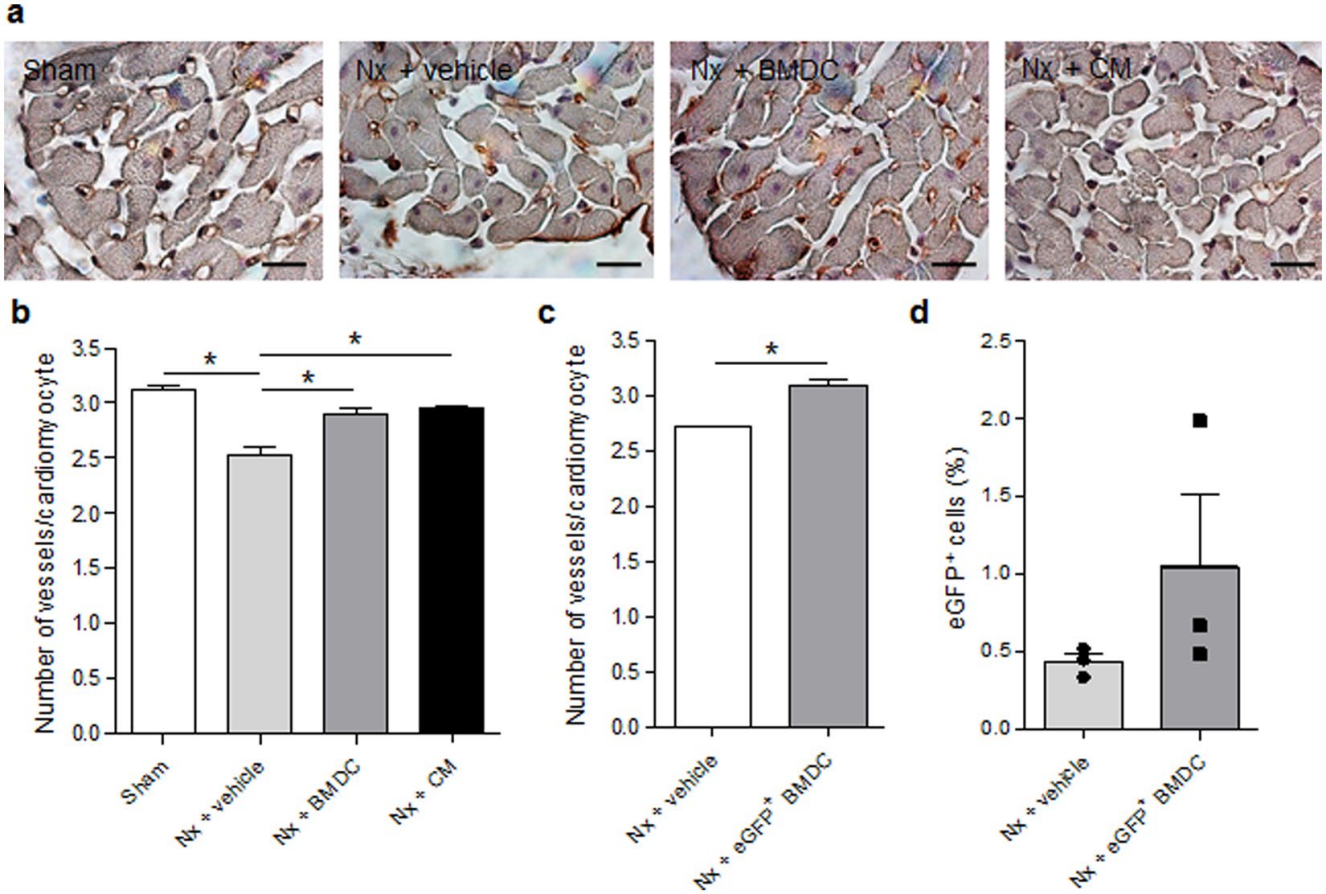
The capillary density in the heart. The number of vessels per cardiomyocyte in the left ventricle was determined 14 days after the sham operation (Sham), 5/6 nephrectomy (Nx), and treatment with vehicle, bone marrow-derived cells (BMDC, 30 × 10^6^ cells per week) or BMDC-conditioned medium (CM, 1 mg protein per week) in Sprague-Dawley (**a–b**) and Lewis rats (**c–d**). (**a**) Capillaries were identified using immunostaining with isolectin B4 (Magnification: 100x, bar: 20 µm), and (**a**) capillary density was expressed as the number of vessels per cardiomyocyte. n = 18–19 for the Sham and vehicle-treated Nx; n = 7–8 for the BMDC- and CM-treated Nx groups. (**a**) Capillary density analysis in Lewis rats after treatment with fluorescent BMDCs that were isolated from transgenic eGFP-Lewis rats. (**a**) Percentage of engrafted eGFP^+^ cells that were analyzed in fresh heart tissue using flow cytometry. n = 3. Results are expressed as a mean ± SEM, *p < 0.05 vs Nx + vehicle.

**Figure 2:**
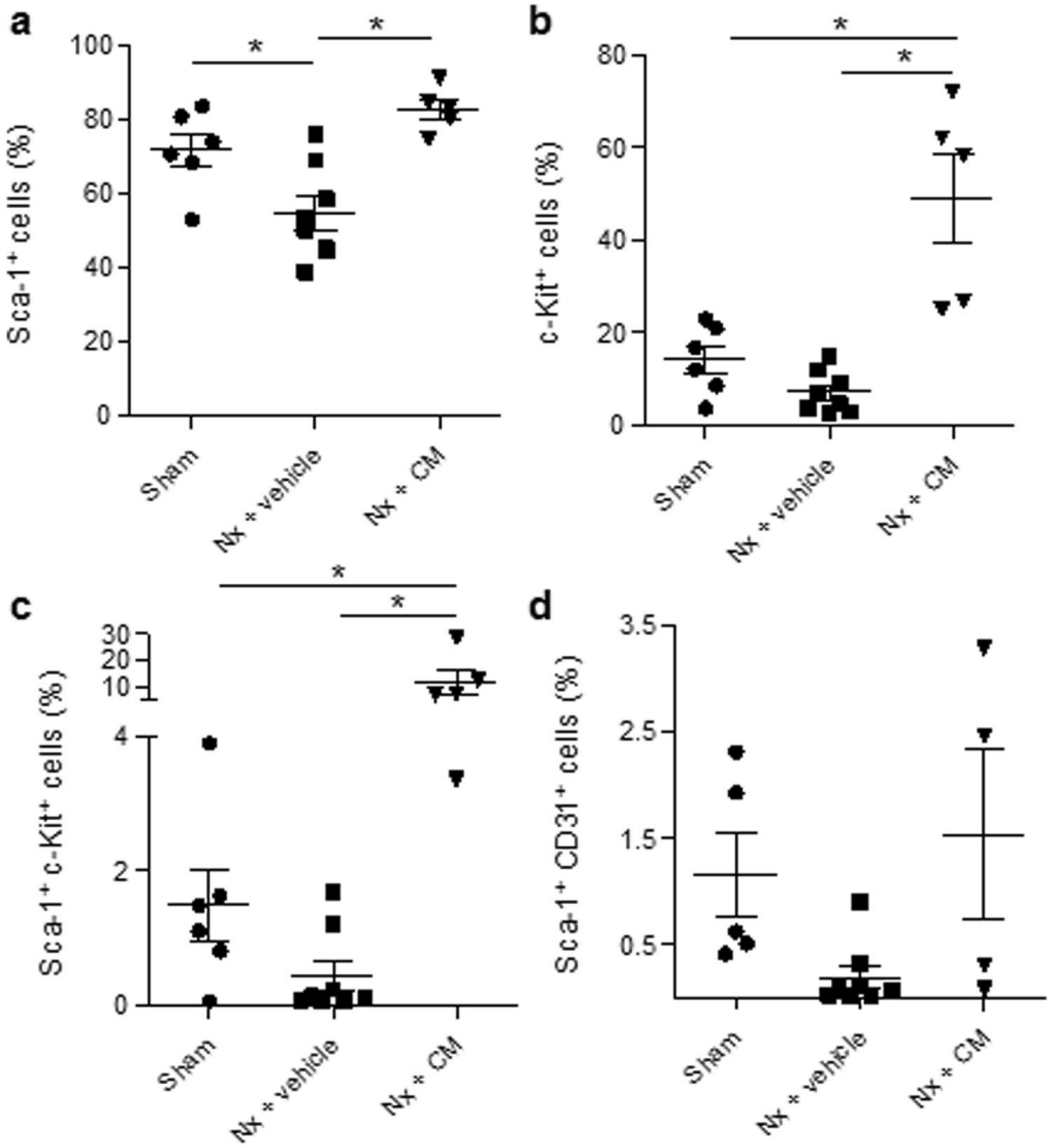
Circulating stem and progenitor cells. The number of endogenous bone marrow-derived stem and progenitor cells was determined in peripheral whole blood using flow cytometry 14 days after surgery/treatment. Hematopoietic stem cells were characterized via the surface expression of stem cell antigen−1 (Sca−1) (**a**), cKit (**b**), or both (**c**). (**d**) Sca-1^+^ cells were further analyzed for the expression of CD31, a marker of endothelial differentiation of progenitor cells. Results are expressed as the mean ± SEM, *p < 0.05 using one-way analysis of variance and post hoc Tukey’s test, n = 5–8. Sham: Sham-operated rats; Nx: 5/6 nephrectomy; BMDCs: bone marrow-derived cells (30 × 10^6^ per week); CM: BMDC-conditioned medium (1 mg protein per week).

Confirming our previous results, we found that experimental uremia, i.e., 5/6 nephrectomy (Nx), induces a 20% reduction in heart capillary density compared with a sham operation, as observed by the reduced number of capillaries per cardiomyocyte stained with an endothelial cell marker 14 days after surgery (Fig. 1a–b). This effect was associated with a decreased number of circulating stem and progenitor cells, identified by the expression of the hematopoietic stem cell marker cKit (CD117) and stem cell antigen-1 (Sca-1) (Fig. 2a–c), but not with differences in the number of Sca-1^+^ cells expressing the endothelial cell marker CD31 (Fig. 2d), as evidenced by flow cytometry of whole blood. In an attempt to replenish the stem and progenitor pool in uremic animals, we treated rats with 30 × 10^6^ BMDCs (a pool of whole bone marrow-derived cells) once a week. This treatment led to restoration of capillary density, as seen in Fig. 1a–b. To track possible cell engraftment *in vivo*, we induced Nx in Lewis rats, and these animals were treated with BMDCs that were isolated from transgenic eGFP-Lewis rats. Two weeks after injecting the rats, we harvested and analyzed the tissues using direct fluorescence via flow cytometry (Fig. 1d) or fluorescence microscopy (data not shown). Even though the protective effects of BMDCs on heart vascularization were confirmed (capillary density increased by 15%, Fig. 1c), no significant engraftment of cells into the heart was found, as seen by the minimal fluorescence threshold just above tissue autofluorescence (Fig. 1d).

Since these findings suggest an endocrine mechanism of action and the importance of released factors in the circulation instead of engraftment into the heart and differentiation into vascular cells, uremic Sprague Dawley rats were treated with CM. CM is characterized by the presence of stem cell-derived secreted factors that include growth factors and a variety of different cytokines, as analyzed with a cytokine array and enzyme-linked immunosorbent assay (Table 1). This treatment not only resulted in similar recovery in capillary density to that observed in uremic animals treated with BMDCs (Fig. 1a–b), but also in a significant increase in circulating Sca-1^+^ and cKit^+^ cells (Fig. 2a–c), as analyzed with flow cytometry. However, serum levels of stromal cell-derived factor-1 (SDF-1) (1,174.07 ± 86.09 vs. 1,182.44 ± 57.92 pg/mL, Nx + vehicle vs. Nx + CM; mean ± SEM, n = 7) and stem cell factor (SCF) (258.8 ± 22.4 vs. 273.5 ± 15.0 pg/ml, Nx + vehicle vs. Nx + CM; mean ± SEM, n = 7), which are both mobilizing factors for endogenous BMDCs (stem and progenitor cells), did not alter in Nx rats after treatment with CM.

**Table 1:** Cytokine profile of BMDC-conditioned medium.

Category	Description
Chemokines	CINC-1, CINC-2, MCP-1, SDF-1
Other cytokines	TNFα, SCF, G-CSF
Growth factors	VEGF-A, agrin, bNGF
ECM and ECM processing	TCK-1, TIMP-1, MMP8

Cytokines were detected by antibody array or ELISA. BMDC: bone marrow-derived cells; bNGF: *beta nerve growth factor;* CINC: cytokine-induced neutrophil chemoattractant; ECM: extracellular membrane; G-CSF: granulocyte-colony stimulating factor; MCP-1: Monocyte chemoattractant protein-1; MMP8: matrix metalloproteinase-8; SCF: stem cell factor; SDF-1: stromal cell-derived factor-1; *TCK-1: t*hymus chemokine-1; TIMP-1: tissue inhibitor of metalloproteinase-1; TNFα: tumor necrosis factor α; VEGF-A: vascular endothelial growth factor-A.

**Figure 3:**
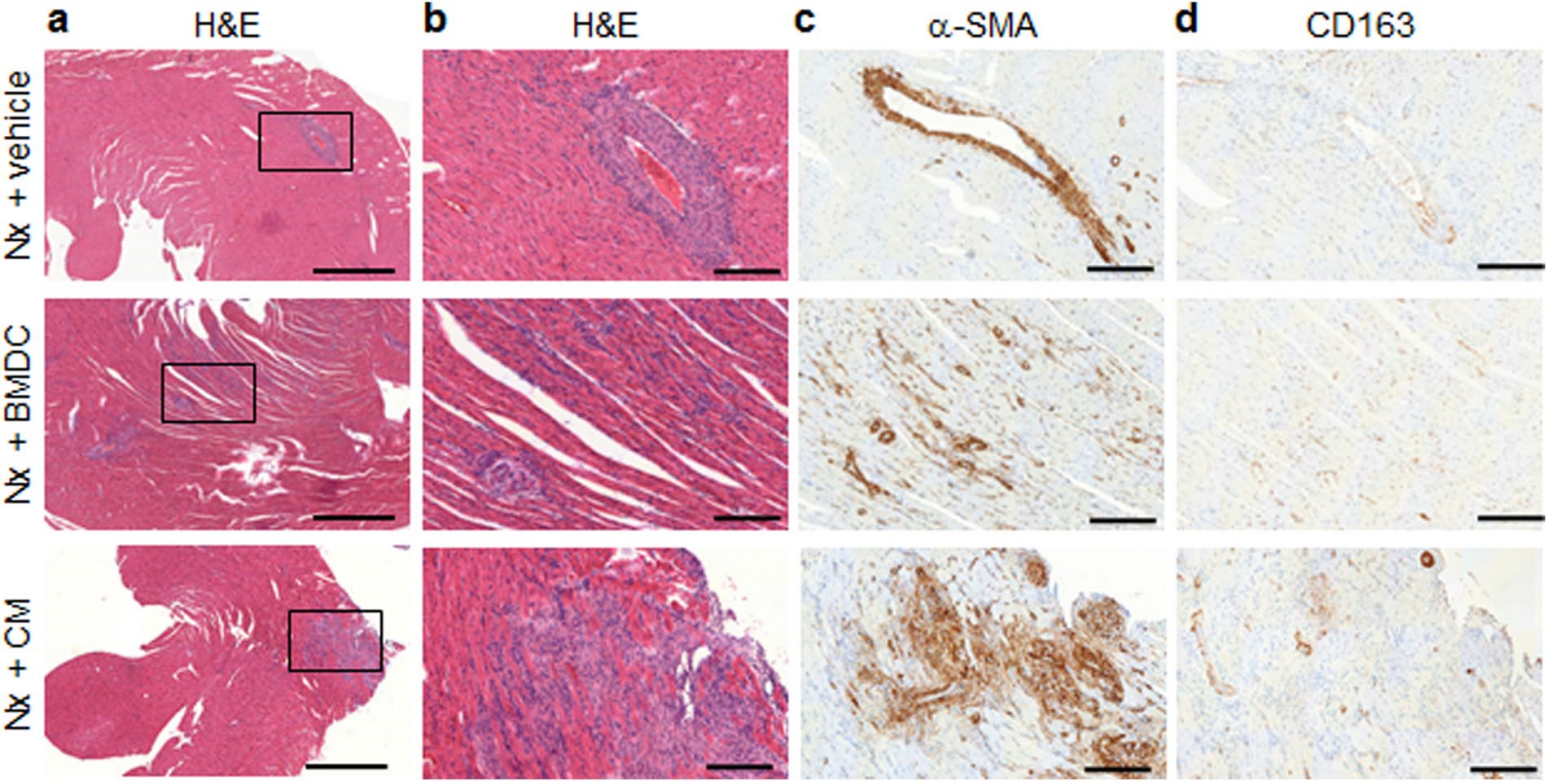
Recruitment and retention of endogenous circulating cells in the heart. Histological and immunohistochemical analyses were performed in hearts 14 days after surgery/treatment. (**a–b**) Hematoxylin-eosin (H&E) staining showed the perivascular location of cell infiltrate in all Nx groups, while a more diffuse, interstitial location was found only in BMDC- and CM-treated Nx rats. Bar: 1 mm in a, and 200 µm in (**b**). Immunohistochemical detections of (**c**) α-smooth muscle actin (αSMA), and (**d**) CD163, a macrophage marker. Bar: 200 µm). Nx: 5/6 nephrectomy; BMDCs: bone marrow-derived cells (30 × 10^6^ per week); CM: BMDC-conditioned medium (1 mg protein per week).

Parallel to the mobilization of cells from bone marrow into the bloodstream, CM induced accumulation of circulating endogenous BMDCs within the heart, an effect that was also observed with BMDC treatment, as evidenced by hematoxylin-eosin (H&E) staining (Fig. 3a–b). Compared with vehicle-treated Nx rats, cell infiltrate in the vessel wall of medium and large vessels was frequently present in BMDC- and CM-treated animals. In addition to the perivascular location, more diffuse, interstitial cell infiltrates were also observed. These cells were not positive for CD34, a marker of nonhematopoietic cell type, including vascular endothelial progenitor cells (Supplemental Fig. 1). Only a few isolated macrophages (Fig. 3d) and T lymphocytes were detected with immunohistochemistry staining, but no granulocytes were seen (data not shown). Moreover, there was no evidence of tissue mineralization by von Kossa staining (Supplemental Fig. 2). However, the strong expression of α-smooth muscle actin (α-SMA), evidenced using immunohistochemistry (Fig. 3c), suggests that these recruited cells adopted a myofibroblast phenotype.

**Figure 4:**
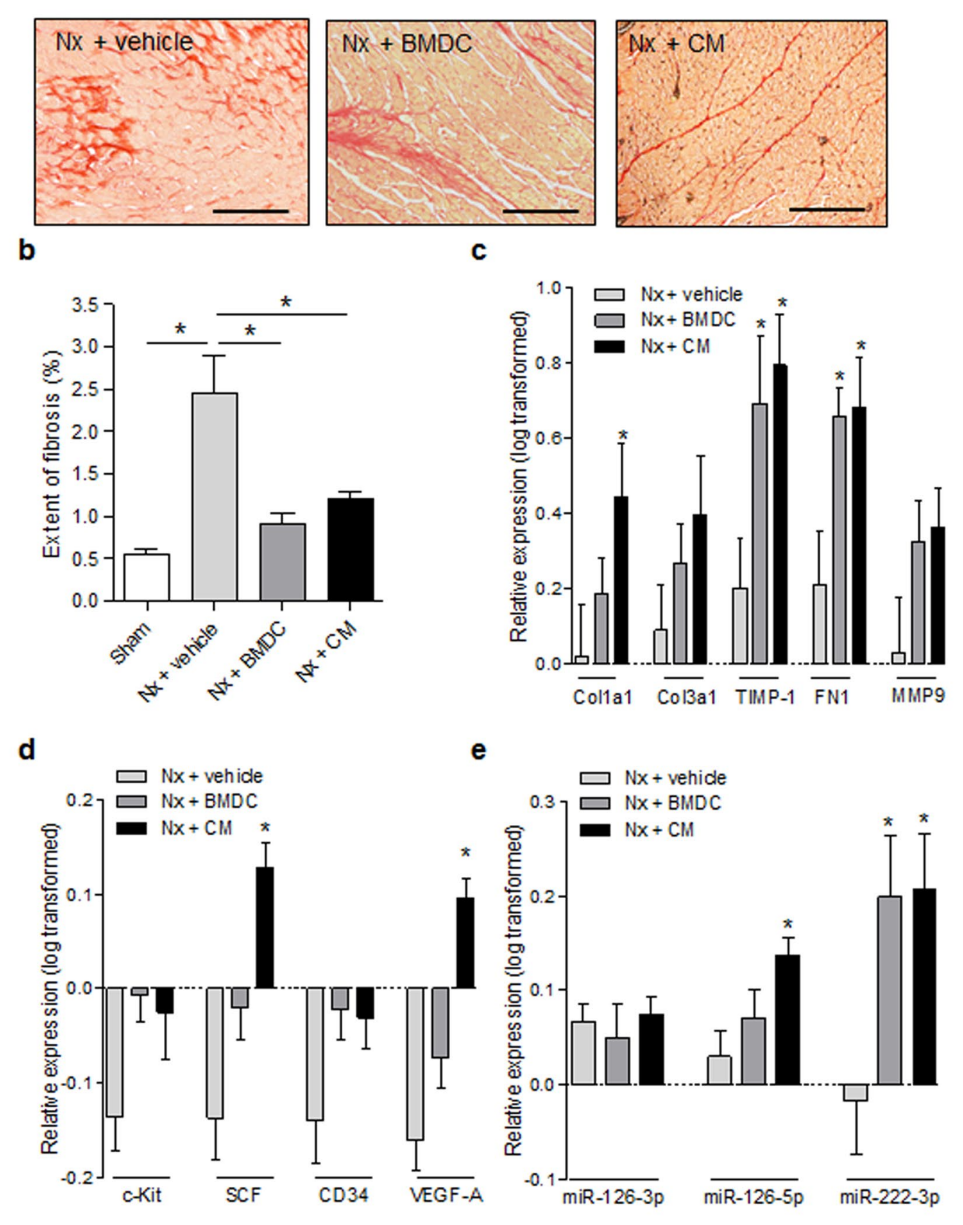
Interstitial fibrosis, extracellular matrix remodeling and expression profile of angiogenesis-related genes and miRNAs. Analyses were performed 14 days after surgery/treatment. (**a**) Visualization of collagen deposition after picrosirius staining (red). Bar: 200 µm. (**b**) Quantification of the extent of fibrosis. n = 16–17 for Sham and Nx + vehicle; n = 8 and 12 for the BMDC- and CM-treated groups, respectively. (**c–d**) Expression profile of extracellular matrix- and angiogenesis-related genes, respectively. (**e**) Expression profile of miRNA. Expression fold-change relative to Sham was analyzed with real-time polymerase chain reaction. n = 4–6. Results are expressed as the mean ± SEM, *p < 0.05 vs. the Nx + vehicle. Sham: Sham-operated rats; Nx: 5/6 nephrectomy; BMDCs: bone marrow derived cells (30 × 10^6^ per week); CM: BMDC-conditioned medium (1 mg protein per week); Col1a1: α-1 type I collagen; Col3a1: α-1 type III collagen; TIMP-1: tissue inhibitor of metalloproteinases 1; FN1: fibronectin 1; MMP9: matrix metallopeptidase 9; miRNA: micro RNA; SCF: stem cell factor (c-Kit ligand); VEGF-A: vascular endothelial growth factor-A.

The presence of these cells is consistent with their role in extracellular matrix (ECM) remodeling and in the cardiac repair process. Consistently, we observed an increased expression of ECM-associated genes in BMDC- and CM-treated rats compared with vehicle-treated animals (Fig. 4c). However, the BMDC and CM treatments led to a significant reduction in the extent of fibrosis (determined using picrosirius staining) in Nx rats compared with vehicle-treated rats (Fig. 4a–b), indicating beneficial remodeling and repair instead of fibrosis formation.

Even though these infiltrate cells do not incorporate within the forming vasculature, they support neovascularization by inducing the expression of pro-angiogenic factors as given in Fig. 4d. Table 2 shows the expression profile of genes involved in neovascularization and cardiac repair in response to BMDC- and CM-treatments. In addition, miRNA expression analysis (Fig. 4e) evidenced an increased expression of miR-126-5p, an endothelial cell-specific regulator of angiogenesis and vascular integrity^29, 30^. Another important miRNA with validated role in angiogenesis is miR-222. Even though its overexpression in endothelial cells seems to inhibit angiogenesis, in cardiac tissue miR-222 has been reported to modulate important physiological function in cardiac stem cells, as well as in conferring protection against adverse remodeling (e.g. decreased cardiac fibrosis) and dysfunction after heart injury^31, 32^.

**Table 2:** Expression profile of genes involved in cardiac repair mechanisms in response to BMDC- and CM-treatment.

Mechanisms of action	Mediators
Neovascularization/wound healing	MCP-1, IL-6, VEGF-A, c-Kit/SCF, MMP9, FN1, CD34
Inflammatory modulation	MCP-1, IL-6, ICAM-1
Anti-remodeling	MMP9, TIMP-1

BMDC: bone marrow-derived cells; CM: conditioned medium; CD34: hematopoietic progenitor cell antigen 1; c-Kit: SCF receptor; FN1: fibronectin 1; ICAM-1: intercellular adhesion molecule-1; IL-6: interleukin-6; MCP-1: Monocyte chemoattractant protein-1; MMP9: matrix metalloproteinase-9; SCF: stem cell factor; TIMP-1: tissue inhibitor of metalloproteinase-1; VEGF-A: vascular endothelial growth factor-A.

**Figure 5:**
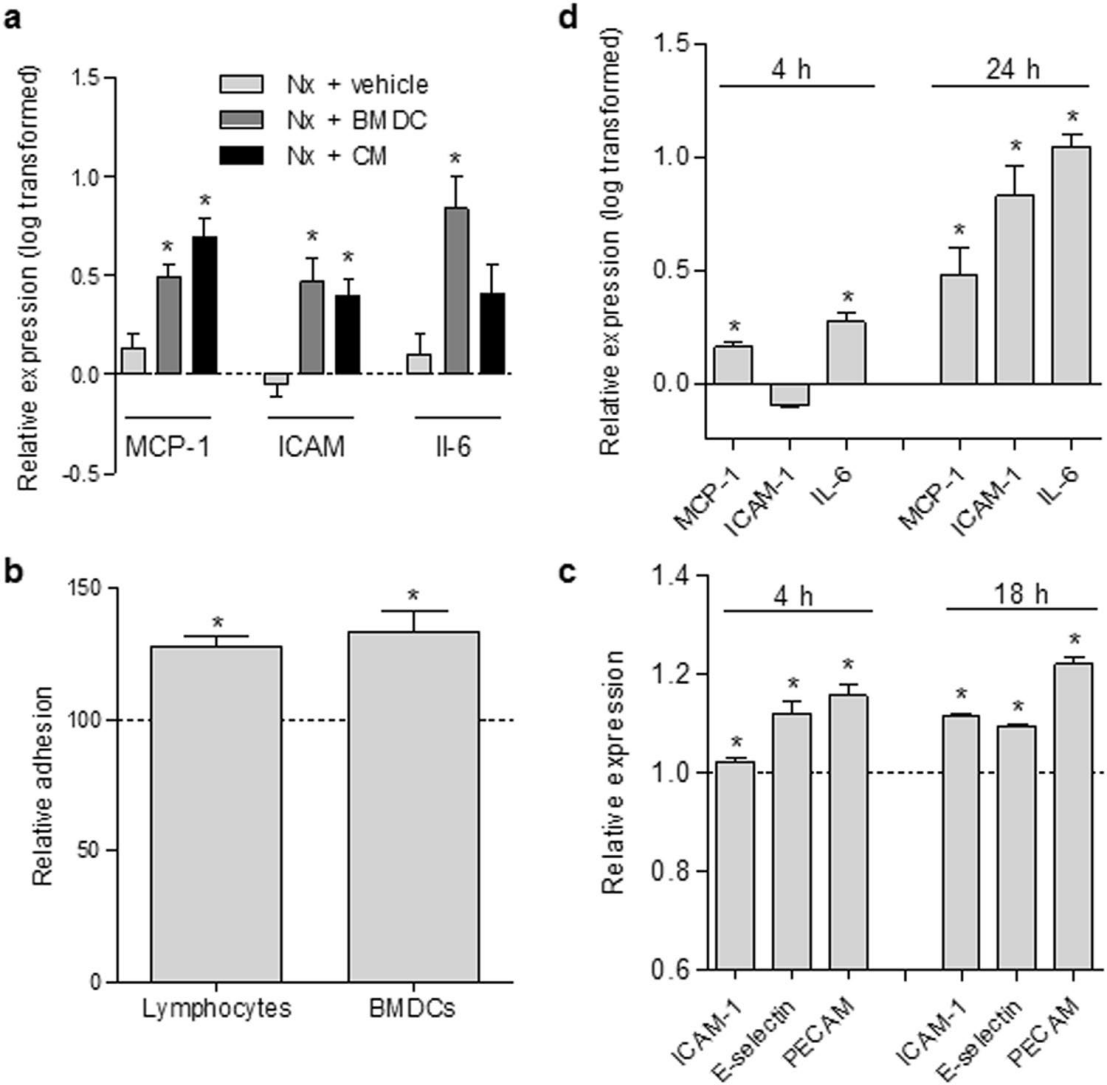
Pro-adhesive phenotype induced by CM. (**a**) Gene expression profile of the hearts 14 days after surgery/treatment. Expression fold-change relative to Sham was analyzed with real-time polymerase chain reaction using 18 S as reference gene. Results are expressed as the mean ± SEM, *p < 0.05 vs. Nx + vehicle, n = 4–6. (**b**) Endothelial adhesion assay. Incubation with CM rendered endothelial cells more adhesive to leukocytes and BMDCs. n = 11–12. (**c**) Surface expression of endothelial adhesion molecules relative to control, determined using flow cytometry 4 and 18 h after exposure to CM; n = 3–6. (**d**) Gene expression profile of endothelial cells that were incubated with CM for 4 and 24 h. Expression fold-change of CM compared with control was determined by real-time polymerase chain reaction, using 18 S as the reference gene. n = 4. From B–D, results are expressed as the mean ± SEM, *p < 0.05 vs. control medium (serum free DMEM); CM: BMDC-conditioned medium (serum-free). Sham: sham-operated rats; Nx: 5/6 nephrectomy; BMDCs: bone marrow-derived cells (30 × 10^6^ per week); CM: BMDC-conditioned medium (1 mg protein per week); ICAM-1: intercellular adhesion molecule-1; IL6: interleukin 6; MCP-1: monocyte-chemoattractant protein-1.

Regarding the recruitment and mobilization mechanisms, BMDC and CM administration to Nx rats upregulated the cardiac expression of inflammatory genes such as interleukin-6 (IL-6), monocyte chemoattractant protein-1 (MCP-1/CCL2), and intercellular adhesion molecule-1 (ICAM-1) (Fig. 5a). Moreover, incubation of endothelial cells with CM for 4 h increased endothelial adhesion to the BMDCs as well as mature leukocytes (lymphocytes and monocytes) compared with the control medium in an *in vitro* adhesion assay (Fig. 5b). Similar to that observed in heart tissues, this effect may be attributed to the increased expression of endothelial adhesion molecules such as ICAM-1, E-selectin (ELAM-1/CD62E), and PECAM-1 (CD31), evaluated using flow cytometry 4 and 18 h after incubation with CM (Fig. 5c). In addition, the gene expression analysis revealed that there was an increased expression of MCP-1 and IL-6 after CM treatment (Fig. 5d).

**Figure 6:**
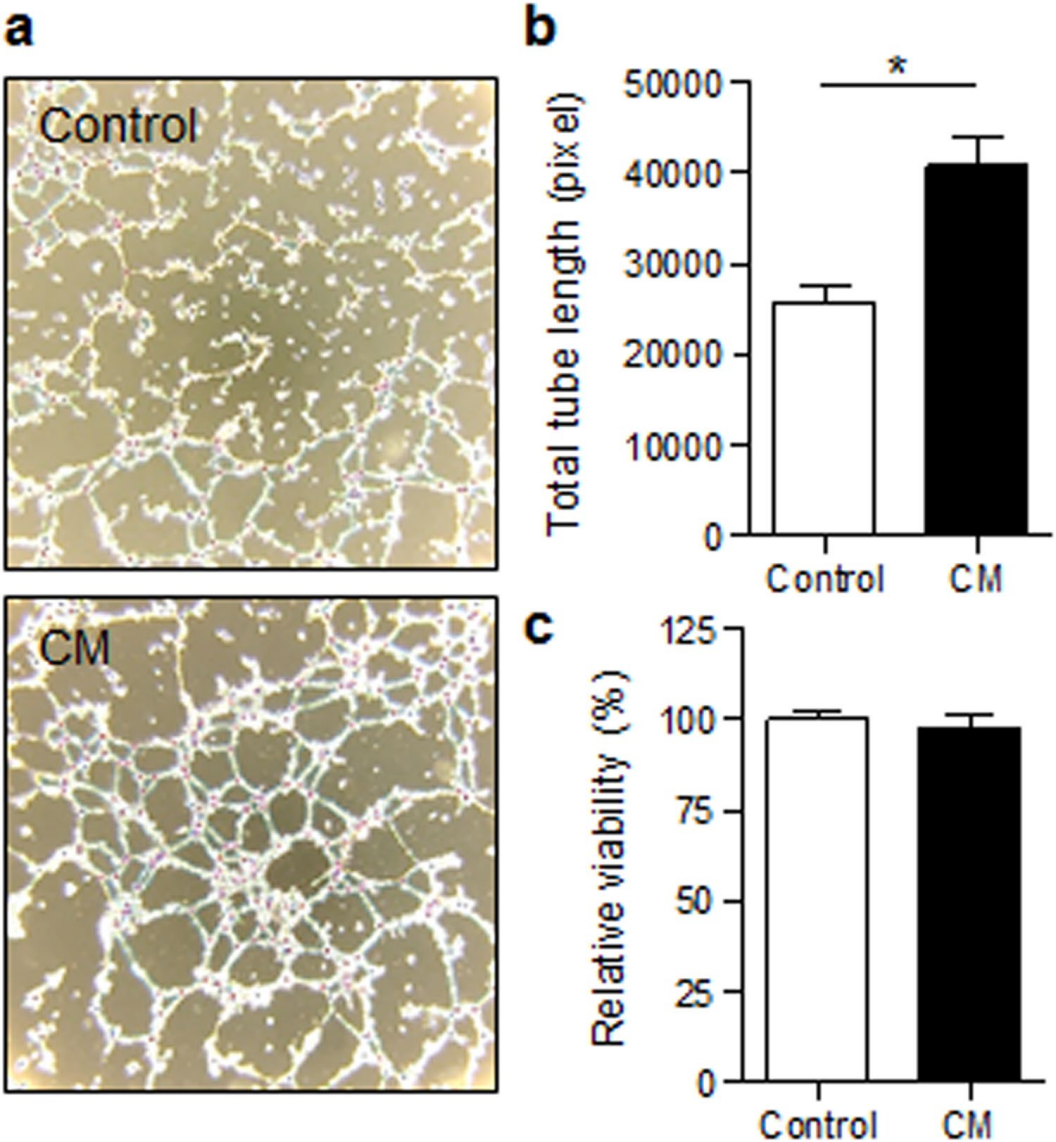
Pro-angiogenic effects of CM on endothelial cells. (**a–b**) *In vitro* formation of capillary-like tubes by endothelial cells on a thin, polymerized layer of matrigel. Analysis was performed 17 h after incubation using a control medium or CM. (**a**) Representative images of tube-like structures. Magnification: 4x; (**b**) Determination of total tube length using Image J Software; n = 4 for control, n = 8 for CM. (**c**) Cell viability, determined with an MTT assay 24 h after incubation with control medium or CM; n = 8 for control, n = 4 for CM. Results are expressed as the mean ± SEM, *p < 0.05 vs. control medium (serum-free DMEM); CM: BMDC-conditioned medium (serum free).

Finally, to explore the proangiogenic and vascular repair activity of CM, an endothelial tube formation assay was performed. As shown in Fig. 6a–b, compared with the control condition, in which tubes were mostly incomplete and most cells were either isolated or aggregated in small clumps (upper panel), in the CM condition, the endothelial cells formed true capillary-like structures with much longer tubes (lower panel). This effect seemed to be independent of cell viability, since no differences could be observed between the groups (both without fetal calf serum) in the MTT assay (Fig. 6c).

**Figure 7:**
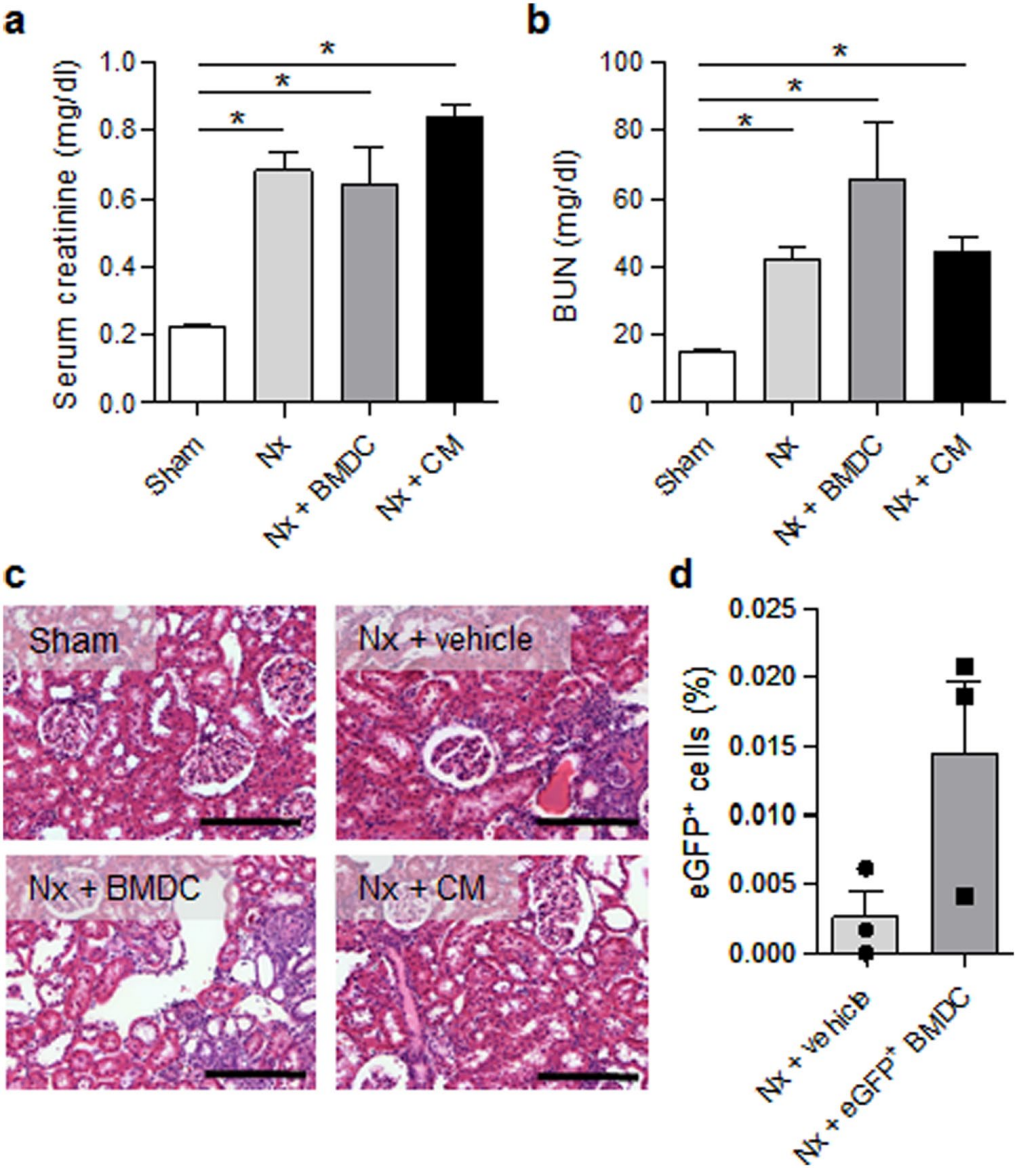
Renal parameters. Functional and histological changes of the kidney were determined 14 days after surgery/treatment. (**a**) Serum creatinine. (**b**) Blood urea nitrogen (BUN). n = 16–19 for the Sham and Nx + vehicle groups; n = 7–8 for the BMDC- and CM-treated Nx groups. (**c**) Hematoxylin-eosin staining. Bar: 400 µm a-c) Sprague-Dawley rats. (**d**) Percentage of engrafted eGFP^+^ cells that were analyzed in fresh kidney tissue with flow cytometry in Lewis rats. n = 3. Results are expressed as the mean ± SEM, *p < 0.05 using one-way analysis of variance and post hoc Tukey’s test. Sham: Sham-operated rats; Nx: 5/6 nephrectomy; BMDCs: bone marrow-derived cells (30 × 10^6^ per week); CM: BMDC-conditioned medium (1 mg protein per week).

Regarding renal parameters, compared with vehicle, treatment with BMDC or CM ameliorated neither the renal function nor renal histology. Here we could observe a fast onset of uremia (14 days after surgery) with typical functional, biochemical signs of renal failure including increased serum creatinine and blood urea nitrogen (BUN) levels (Fig. 7a–b), increased urine volume and decreased creatinine clearance (Table 3). Structural and morphological changes include glomerular and tubular damage (Fig. 7c, Table 3). All Nx rats show sustained proteinuria and protein casts within the tubules. Vehicle-treated animals display classic atrophic tubules with thick tubular basement membrane and simplified epithelium, while tubules in BMDC- and CM-treated animals are dilated and enlarged. It may occur in consequence of renal ischemia and interstitial inflammation and/or fibrosis, which can be observed in all studied groups, except in Sham-operated animals. Table 3 summarizes general and laboratory parameters determined 14 days after surgery/treatment.

**Table 3:** Summary of parameter values in the 5/6 Nephrectomy (Nx) model 14 days after treatment with BMDCs, CM or vehicle.

Parameter	Sham	Nx + vehicle	Nx + BMDC	Nx + CM
	n = 16	n = 19	n = 8	n = 8
*General parameters*				
Weight gain (g)	147 ± 30	−3 ± 9*	−22 ± 18*	−22 ± 15*
Heart/body ratio	0.34 ± 0.03	0.44 ± 0.01*	0.45 ± 0.02*	0.41 ± 0.01*
*Renal function parameters*				
Creatinine clearance (ml/min/100 g)	0.93 ± 0.02	0.30 ± 0.03*	0.29 ± 0.04*	0.23 ± 0.02*
Urine protein (mg/24 h)	32 ± 2	82 ± 22*	95 ± 36*	76 ± 22*
*Fluid balance*				
H_2_O intake (ml/24 h)	35 ± 2	56 ± 4*	55 ± 4*	70 ± 6*
Urine volume (ml/24 h)	19 ± 2	42 ± 4*	34 ± 5*	47 ± 5*
*Electrolyte balance*				
Serum Na^+^ (mmol/l)	142.5 ± 0.4	142.5 ± 0.5	141.5 ± 1.9	141.3 ± 0.4
Serum K^+^ (mmol/l)	5.3 ± 0.1	5.2 ± 0.1	5.3 ± 0.3	4.9 ± 0.2
FE Na (%)	0.3 ± 0.02	1.2 ± 0.15*	1.1 ± 0.25*	1.6 ± 0.12*
FE K (%)	19.2 ± 0.6	78.0 ± 7.5*	80.0 ± 14.4*	101.6 ± 7.4*
*Serum parameters*				
Calcium (mg/dl)**	2.5 ± 0.02	2.4 ± 0.05	2.7 ± 0.04	2.8 ± 0.04
Phosphate (mg/dl)**	6.9 ± 0.3	5.6 ± 0.5	5.9 ± 0.4	3.9 ± 0.3*
PTH (pg/ml)***	124 ± 13	233 ± 40*	125 ± 32	87 ± 14^#^
Aldosterone (ng/dl)**	1.3 (0.9–1.4)	23 (12–35)*	40 (35–68)*	100 (49–235)*^#^

Results are mean ± SEM or median (25^th^–75^th^ percentiles). Weight gain: difference (in g) between weight from pre- to post-treatment. *P < 0.05 vs. Sham; ^#^P < 0.05 vs^.^ Vehicle. **n = 6 per group; ***n = 12 for Sham and Nx + vehicle, n = 6 for Nx + BMDC and n = 11 for Nx + CM. FE, fractional excretion; PTH, parathyroid hormone. Sham: Sham-operated rats; BMDCs: bone marrow-derived cells (30 × 10^6^ per week); CM: BMDC-conditioned medium (1 mg protein per week).

Engraftment of exogenous, eGFP-BMDCs was not found in the kidney (Fig. 7d).

## Discussion

**Figure 8:**
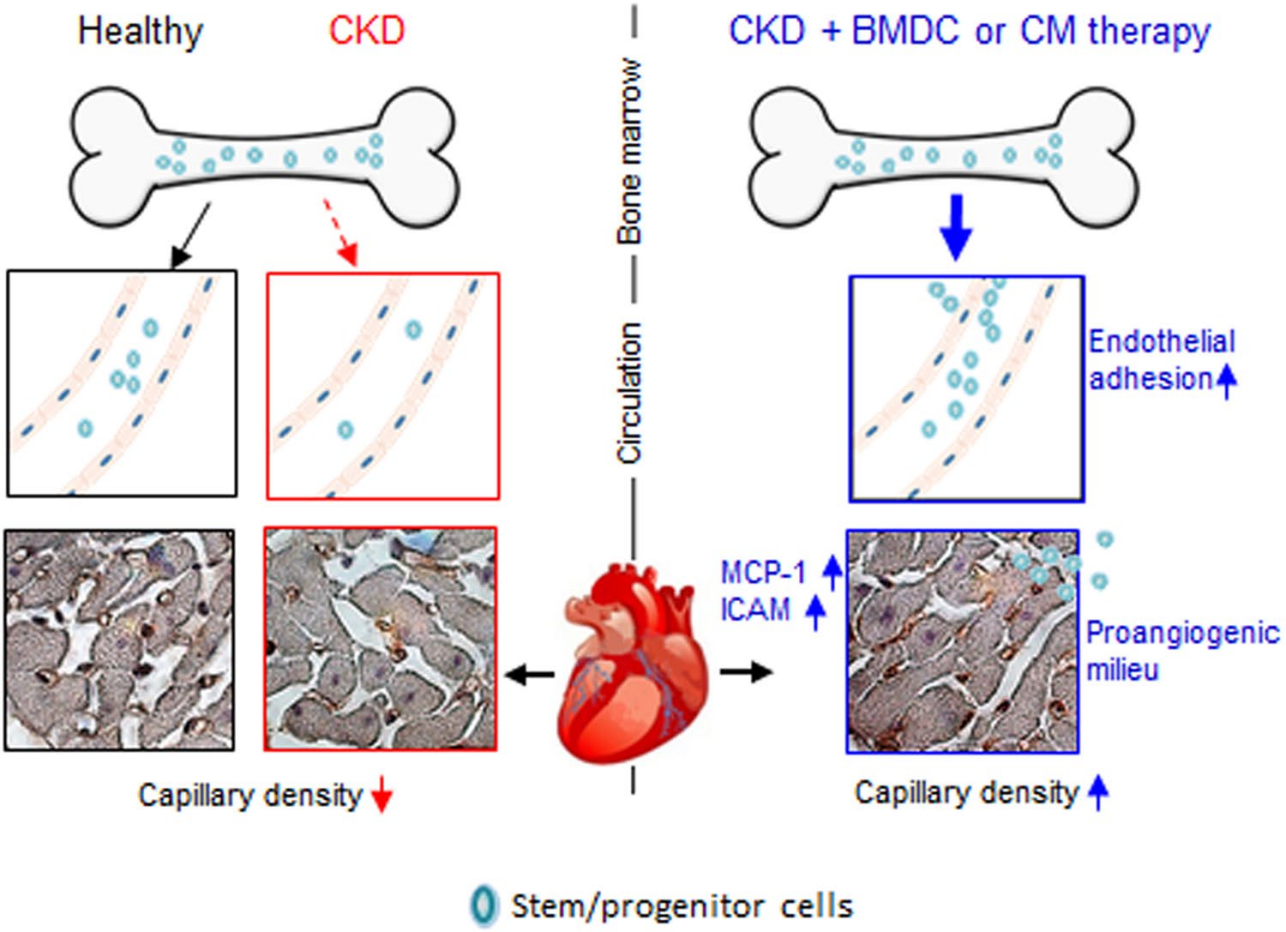
Overview of the processes of cardiac repair induced by BMDC or CM therapy in CKD. During CKD, the number of circulating progenitor cells as well as the capillary density in the heart are decreased. Treatment with exogenous BMDCs or their CM stimulates the endogenous repair mechanisms that include 1) mobilization of endogenous BMDCs from the bone marrow into the blood stream, 2) upregulation of endothelial adhesion factors that facilitate 3) recruitment and infiltration of endogenous circulating cells into the heart, and 4) positive regulation of angiogenesis. BMDCs: bone marrow-derived cells; CKD: chronic kidney disease; CM: BMDC-conditioned medium; MCP-1: monocyte-chemoattractant protein-1; ICAM: intercellular adhesion molecule-1.

Remodeling of the heart is commonly observed in patients with CKD and it is associated with fibrosis, and capillary rarefaction^33^. Impaired angiogenesis plays an important role in ventricular remodeling, heart dysfunction, and subsequent heart failure^4, 5^. Treatment with BMDCs or CM is suggested to induce angiogenesis and has therapeutic effects on both the kidney and heart after injury^17, 19^. In this context, we investigated the effects of BMDCs and their CM on the vascular repair of the heart of uremic rats and observed that both treatments were able to restore vascular density by stimulating endogenous repair mechanisms, as summarized in Fig. 8.

The 5/6 nephrectomy rat model recapitulates many features of human CKD, including impaired number of circulating stem/progenitor and decreased capillary density and increased fibrosis in the heart. As expected from previous experimental studies^17, 18^ and clinical trials^15, 16^, BMDC therapy was successful in preventing capillary rarefaction and fibrosis formation, although no significant engraftment of exogenous cells was found. This indicates the importance of secreted, circulating factors (all of which are found in the CM) rather than cell engraftment for tissue repair.

Cardiac repair is a complex process that involves inflammation, angiogenesis, and remodeling^34^. In addition to the need of resident cells, circulating cells also actively participate in inducing angiogenesis and vasculogenesis. The first step in this process is the mobilization of cells from bone marrow into the circulation^35^. This step may be compromised in Nx rats and CKD patients, as seen in the reduced number of circulating stem and progenitor cells in previous studies^7, 10, 36^. In rats, treatment with CM stimulates mobilization and increases the number of circulating cells that express hematopoietic stem cell markers. Besides metalloproteinases, which are proposed to provide a permissive environment for the egress of cells from the bone marrow^37^, different mobilizing chemokines such as SDF-1, G-CSF and SCF^38^ were found in the CM. A variety of experimental studies suggested that factors that induce mobilization of endogenous BMDCs augment recovery after ischemia, improve neovascularization, and provide beneficial effects *in vivo* ^24^.

The next step after mobilization is the recruitment of cells to the site of the injury. BMDC and CM treatment upregulated the expression of pro-inflammatory genes (MCP-1, IL-6 and ICAM-1) in the heart of Nx rats. It is worth pointing out that the interaction of stem and progenitor cells with the endothelium in the vessel wall mimics similar pathways that are involved in leukocyte mobilization in inflamed tissues37. MCP-1 is well known for the recruitment of mesenchymal cells, monocytes, and macrophages towards endothelial cells in ischemic tissue;^39^ while IL-6, by inducing the expression of the endothelial adhesion molecule ICAM-1, can also lead to the recruitment of cells^37^.

In agreement with these findings, we found that endothelial cells that were treated with CM showed increased expression of adhesion molecules (E-selectin, ICAM-1, and PECAM-1), which are associated with rolling, adhesion, and transmigration of blood cells through the endothelium^35^. ICAM-1 is responsible for firm adhesion of leukocytes to endothelial cells and was closely associated with endothelial progenitor cell entrapment in an animal model of hind limb ischemia^35^. The expression of adhesion molecules in endothelial cells may not only facilitate the recruitment of cells in the heart, but also the release of cells from the bone marrow into the bloodstream. Since the hematopoietic compartment consists of a monolayer of endothelial cells, the same molecules that are important for transmigration from the circulation to the injured site are also important for the attachment and transmigration of these cells through the bone marrow endothelium to the circulation^37^.

The infiltration of endogenous cells may participate in organ regeneration. However, regeneration depends on the intrinsic character of the recruited cells and the microenvironment that is present at the site of delivery. This microenvironment often has features of a healing wound, including inflammatory cells, neovasculature, and pro-fibrotic cytokines^40^. In our study, even though only few macrophages and lymphocytes could be seen in the hearts of Nx animals after 14 days of treatment, we could not exclude the possibility of the presence of an exacerbated inflammatory response at earlier time points. This is important because inflammation always accompanies angiogenesis, as seen by collateral growth in ischemic organs^35^. In this context, upregulation of the inflammatory genes that was observed here might contribute to inflammation and angiogenesis in the heart by recruiting circulating cells. These in turn are pivotal for supplying the damaged tissue with cytokines that are responsible for the expression of adhesion molecules and subsequent recruitment of endogenous BMDCs that act as vasculogenic cells by releasing proangiogenic factors, even without incorporating within the forming vasculature^23, 24, 34^. Interestingly, aldosterone has appeared to actively participate in neovascularization not only due to its pro-inflammatory action, thereby regulating the expression of MCP-1 and ICAM-1 and favoring the adhesion of immune cells, but also by regulating the secretion of angiogenic molecules – especially VEGF-A – by these cells^41–43^.

Treatment with BMDCs or CM favors a proangiogenic milieu, as seen by the increased number of capillaries per cardiomyocyte in BMDC or CM-treated rats compared with vehicle-treated rats. MCP-1 and IL-6, which were upregulated due to these treatments, may have contributed to these effects since they are proangiogenic cytokines that induce angiogenesis both *in vitro* and *in vivo* ^44–47^.

We found that infiltrated cells were positive for αSMA, a contractile protein that is highly expressed by myofibroblasts. These cells are not normally found in the healthy myocardium, but are the most prevalent cell type in the infarct scar, given that transient activation of myofibroblasts is part of the normal wound healing process^48, 49^. Despite the cell infiltrate with myofibroblast-like phenotype and high aldosterone levels (pro-fibrotic effects)^41, 42^, the hearts of rats that were treated with BMDC or CM displayed a diminished extent of fibrosis compared with those treated with the vehicle, suggesting that the activation of these cells should be transient and contributes to beneficial remodeling instead of fibrosis formation. Moreover, reduced fibrosis formation observed in these animals might also be partially due to the preservation of the microvascular integrity by BMDC and CM treatments^50^.

Furthermore, there were no signs of tissue mineralization within cell infiltrate, in the myocardium or in blood vessels after treatment with BMDCs or CM. This is particularly important considering that inflammation and mineral and bone disorders, inherent complications of CKD, may induce bone marrow-derived cells, including hematopoietic and mesenchymal stem cells and even endothelial progenitor cells, to differentiate towards an osteogenic phenotype and function as circulating calcifying cells^51, 52^. In this context, factors like PTH, phosphate levels and IL-6 could favor ectopic calcifications. On the other hand, however, these are well-known stimuli for hematopoietic stem cell expansion inside their bone marrow niche^51, 53^.

The vasculoprotective effects that were observed here may not rely exclusively on the increased mobilization of circulating cells, since the CM also exerts direct effects on the endothelial cells and vasculature, as shown by us and others^11, 19^. In addition to rendering endothelial cells more adhesive to BMDCs and leukocytes by increasing the expression of adhesion molecules, the CM also stimulates endothelial tube formation, thus underlining its proangiogenic effects. Several factors present in the CM (such as MCP-1 and VEGF-A; Table 1) as well as the factors that are upregulated in endothelial cells by CM treatment (such as MCP-1 and IL-6) could be responsible for this proangiogenic response^44, 47, 54^. One factor, such as MCP-1 or IL-6, may contribute to more than one process, leading to angiogenesis and inflammation. Moreover, in CM, various factors may act together to promote tissue regeneration^54^.

Here we applied healthy BMDCs to Nx rats and to generate CM. Considering clinical application, autologous cells are required to avoid immunologic reactions. However, the presence of uremia is known to negatively influence BMDC availability and function and alter their cytokine production^10, 55, 56^, which could limit the therapeutic possibilities. However, van Koppen *et al*. have shown that BMDCs that are isolated from uremic rats are also able to reduce progression of kidney failure, although in a less effective manner than that of healthy BMDCs^57^.

Regarding renal parameters, although BMDC and CM therapies have been shown to have beneficial effects on different models of kidney disease^18, 19, 57, 58^, no improvement was noted in our study. There are several explanations for this discrepancy, such as the type and amount of applied cells, method of application (systemic vs. intrarenal), processing, and cytokine levels (concentration factor) of the CM^54, 59^. Here, however, the rapid onset of uremia (less than 14 days after surgery), fast progression and renal lesion severity may have hindered therapeutic efficacy. Moreover, the time point of the analysis is also very important, because the benefits associated with the BMDC therapy relied mostly on retardation instead of complete prevention of CKD progression. Van Koppen *et al*. have observed reduced progression of kidney failure in Nx rats only five weeks after therapy with BMDC or CM; noting that, in their study, therapy started four weeks after surgery, i.e. when progression rates are already very low^60, 61^. Furthermore, we cannot exclude that, under our experimental conditions, these treatments have negatively affected renal function/morphology by, for example, exacerbating renal inflammation. Nevertheless, we analyzed the rats 14 days after surgery, which is an early time to evaluate differences in progression; however, it is a good time to show that the proposed therapy has beneficial effects on CKD-associated heart remodeling, independent of changes in renal function.

Regenerative medicine is an alternative to ameliorate CKD-associated heart remodeling and even renal disease progression. Cell-based therapy has been extensively discussed due to its contradictory findings^59, 62, 63^. Multiple factors such as the heterogeneity of cell types, isolation method, and even autotransplantation of non-functional cells could have interfered with these results^59, 64^. The use of a cell-free treatment (i.e., CM) would be an advantageous alternative in regenerative medicine, since it can be manufactured using standardized methods of production, and it can be validated for commercial use^54^.

In summary, our findings indicate that treatment with exogenous BMDCs and CM had beneficial effects and avoided capillary rarefaction in an animal model of CKD by stimulating endogenous repair mechanisms. Strategies aimed at boosting the endogenous reparative potential as described here would at least slow down the progression of the disease, allowing structural and functional reorganization and restoration of the damaged tissue.

## Methods

Experiments were approved by a governmental committee on animal welfare *“Landesamt für Natur, Umwelt und Verbraucherschutz Nordrhein-Westfalen” (84-0*2*.04.2012.A298)* and performed in accordance with national animal protection guidelines.

Detailed methods are given as Supplemental Data.

### Animal model

For the animal model of CKD, renal injury was induced in 260–300 g, healthy male Sprague-Dawley rats by 5/6 nephrectomy (Nx) as described before^65^. After surgery, rats were randomized into different groups: Nx + vehicle rats received saline injection (i.p.) once a week; Nx + BMDC rats received 30 × 10^6^ BMDCs/week (i.v.), isolated from 4-week old, healthy donor rats; Nx + CM rats received 1 mg total protein of CM per week. Sham operation consisted of midline incision and decapsulation of the right kidney. On day 13, rats were individually housed in metabolic cages for 24 h for urine collection. Rats were fed standard diet containing 0.6% phosphorus and 0.6% calcium (Altromin maintenance diet 1324, Lage, Germany). Animals were sacrificed on day 14, blood was collected and hearts were excised and prepared for molecular and histological analyses as described below. Urine and serum were analyzed to assess metabolic parameters.

To analyze engraftment of BMDCs, Nx was performed in 260–300 g male Lewis rats. Animals were randomized into two groups: rats received either vehicle (i.p. saline injection once per week) or 30 × 10^6^ eGFP^+^ BMDCs (i.v. injection once per week). eGFP^+^ BMDCs were isolated from 4-week old, GFP-transgenic Lewis rats. After 14 days, hearts were excised und submitted to flow cytometry analysis as described below.

### BMDC isolation and BMDC-conditioned medium

For the isolation of BMDCs, tibia, femur and humerus were removed from 4-week old, healthy Sprague-Dawley or GFP-transgenic Lewis rats and placed in ice-cold PBS with 10 U/ml heparin as modified from Yang et al^66^. The whole bone marrow was flushed out and filtered using a 70 µm Cell Strainer. After centrifugation, erythrocytes were lysed, and the sample was washed. After cell counting and centrifugation, cells were resuspended in serum free DMEM containing 2 mM L-glutamine, and 50 U/ml each of penicillin/streptomycin at the concentration needed for experiments or stored in liquid nitrogen using 50% fetal calf serum, 40% DMEM and 10% DMSO.

For BMDC conditioned medium (CM), isolated BMDCs were seeded either on a 24-well plate at a concentration of 2 × 10^6^ cells/well (for *in vitro* assays) or in a cell culture flask at a concentration of 60 × 10^6^ cells/5 ml culture medium (for *in vivo* experiments). Cells were incubated with serum free DMEM for 24 h at 37 °C and 5% CO_2_, before they were centrifuged at 1000 × *g* for 6 min. The supernatant was stored at −20 °C. For the treatment of rats, the CM was concentrated using Amicon Ultra-4 Centrifugal Filters (3 K) to a final concentration of 2 mg/ml total protein as modified from van Koppen et al^19^.

### Capillary density

For the staining of capillaries in the heart, Isolectin B4, a marker of endothelial cells, was used as previously described^3^. For visualization of cell borders and cell nuclei, a hematoxylin-eosin staining was performed. Digitized pictures (8 separate high-power fields per section) were taken from the left ventricle by using a Carl Zeiss microscope and the AxioVisonLE Release 4.7.1 software with a 100x magnification. Heart vascularization was determined by counting the number of blood vessels per cardiomyocyte^3^.

### Histological Analysis and assessment of fibrosis

To determine the extent of fibrosis, the collagen in paraffin-embedded tissue sections was stained with Picrosirius red, as previously described^67^. Digitized pictures (8 separate high-power fields per section) were taken from the left ventricle by using a Carl Zeiss microscope and the AxioVisonLE Release 4.7.1 software with a 20x magnification. Extent of fibrosis was calculated using ImageJ software.

Kidney and heart histology by H&E (Roth, Karslruhe, Germany) and von Kossa stainings (Merck, Darmstadt, Germany) were performed according to manufacturers’ instructions.

### Immunohistochemistry

Immunohistochemistry was performed on histological sections of paraffin-embedded tissue samples using the Ventana OptiView IHC Detection Kit following standardized protocols of the manufacturer. The following primary antibodies were used: monoclonal α-smooth muscle actin antibody (αSMA; Cell Marque, clone 1A4) at a ready to use dilution of 0.02 µg/ml; monoclonal CD163 (Cell Marque, clone MRQ-26) at a ready to use dilution of 0.17 µg/ml; and monoclonal CD4 (Ventana, clone SP 35) at a ready to use dilution of 2.5 µg/ml.

### Gene and miRNA expression

Total RNA was isolated from heart tissue stored in RNAlater or EA.hy926 cells directly harvested in RLT-Buffer (RNEasy Mini Kit). The gene expression was then analyzed by real-time PCR using the SYBR Select Master Mix (Applied Biosystems) as described before^67^. The relative gene expression was analyzed using the 2^−ΔΔCt^ method and 18 S as reference gene. Results were log-transformed before statistical analysis. Rat primer sequences are:

MCP-1 forward 5′- gctgctactcattcactggcaa-3′ and reverse 5′-tgctgctggtgattctcttgta-3′; ICAM forward 5′-cgggagatgaatggtacc-3′ and reverse 5′-gcggtaataggtgtaaatgg-3′; Il-6 forward 5′-ttggatggtcttggtccttagcc-3′ and reverse 5′-tcctaccccaacttccaatgctc-3′; Il-10 forward 5′-ctcccctgtgagaataaaagcaag-3′ and reverse 5′-agtgtcacgtaggcttctatgc-3′; 18 S forward 5′-gcggcttaatttgactcaacac-3′ and reverse 5′-agacaaatcgctccaccaacta-3′. Human primer sequences are: MCP-1 forward 5′-tgcagaggctcgcgagcta-3′ and reverse 5′-caggtggtccatggaatcctga-3′; ICAM forward 5′-tgtgaccagcccaagttgtt-3′ and reverse 5′-agtccagtacacggtgagga-3′; IL-6 forward 5′-acatcctcgacggcatctca-3′ and reverse 5′-caccaggcaagtctcctcatt-3′; 18 S forward 5′-ctcaacacgggaaacctcac-3´ and reverse 5′-cgctccaccaactaagaacg-3′.

For analysis of miRNA expression, the following commercial kits were used: mirVana miRNA isolation Kit (Invitrogen), TaqMan Advanced miRNA cDNA Synthesis Kit (Applied Biosystems) and the TaqMan Advanced miRNA Assays: rno-miR-126-3p, rno-miR-126-5p, rno-miR-222-3p and rno-let-7g-5p. The relative expression was analyzed using the 2^−ΔΔCt^ method and rno-let-7g-5p as endogenous control. Results were log-transformed before statistical analysis.

### Cytokine Array

The cytokine array was performed using the Rat Cytokine Antibody Array C2 kit (RayBio) following the manufacturer’s instructions.

### Elisa

Commercial ELISA Kits were used to determine levels of stem cell factor (mouse SCF; R&D Systems), stromal cell derived factor 1 (rat SDF-1; Cloud Clone Corp.), granulocyte-colony stimulating factor (rat G-CSF; CUSABIO) and parathyroid hormone (rat intact PTH; Immutopics) in serum and/or CM. Rat serum aldosterone was determined by chemiluminescent immunoassay technology with an automated LIAISON® analyzer system (DiaSorin Deutschland GmbH, Dietzenbach, Germany).

### Endothelial cell culture

EA.hy926 cells, a human umbilical vein endothelial cell line that expresses highly differentiated functional characteristic of human vascular endothelium^68, 69^, were grown in DMEM containing 5% fetal calf serum, 2 mM L-glutamine, and 50 U/ml each of penicillin/streptomycin at 37 °C in an atmosphere of 5% CO_2_ in air.

For gene expression, cells were cultured in 24-well plate. At 80–90% confluence, cells were treated with CM or serum free DMEM for different periods of time (4 or 24 h). After incubation, endothelial cells were harvested and submitted to RNA extraction and gene analysis as described above. Additional culture conditions and treatments are described below.

### Viability assay

The effects of CM on endothelial cell viability was assessed using MTT assay^70^. In brief, endothelial cells were cultured in a 96-well plate (80–90% confluence, 100 µl medium/well) were treated with CM or serum free DMEM for 24 h. At the end of the incubation time, 5 µl of MTT-solution (5 mg/ml in NaCl 0.9%) was added to each well, and cells were further incubated for 3 h. Medium was removed and cells were solubilized with a lysing solution (100 µl/well; 100 ml 20% SDS, 34 ml N_3_N-dimethylformamide, 16 ml distilled water) overnight. The absorbance was measured at 590 nm in a microplate reader.

### Tube formation assay

In order to analyze the proangiogenic activity of CM, a tube-formation assay was performed using matrigel^71^. A µ-slide Angiogenesis (ibidi) was coated with 10 µl matrigel/well and incubated for 5 h at 37 °C. EA.hy926 cells were seeded on the matrigel at a concentration of 1 × 10^4^/well with either CM or serum free DMEM as control and incubated for 17 h. Pictures were taken with a 4x magnification and analyzed using the Angiogenesis Analyzer for ImageJ.

### Adhesion Assay

Leukocyte- and BMDC-endothelial adhesion was determined as previously described with some minor modifications^72^. In brief, peripheral blood leukocytes were separated from EDTA-blood of healthy volunteers by density gradient centrifugation (Lymphocyte Separation Medium, 1077 density). BMDCs were isolated as described above. The cells were labeled with calcein-AM (3 μM) in phenol red-free RPMI containing 5% fetal calf serum (Washing medium) for 30 min at 37 °C protected from light. Cells were washed twice and resuspended in binding medium (phenol red-free RPMI containing 2% fetal calf serum). The cells were then counted and added (150–300 × 10^3^/well, 100 µl volume) to confluent monolayers of EA.hy926 cells that had been grown in 96-well plates and treated for 4 hours with serum free DMEM or CM. The amount of labeled cells added was assessed by measuring the fluorescence signal (total signal) using a fluorescence spectrometer equipped with a microplate reader (Ex: 485 nm, Em: 530 nm). After 60 or 180 min incubation at 37 °C for leukocytes and BMDCs, respectively, non-adherent cells were removed by washing 2–3 times with pre-warmed washing medium. The fluorescent signal was reassessed by the microplate reader (adherent signal) in the presence of 100 µl binding medium. The percentage of leukocytes adhering to the endothelial monolayer was calculated by the formula: % adherence = (adherent signal/total signal) × 100.

### Flow cytometry

For the analysis of BMDC-engraftment, heart and kidney were mechanically shredded using a scalpel and pressed through a 70 µm Cell Strainer. After washing with PBS, the suspension was processed by density gradient centrifugation (Lymphocyte Separation Medium, 1077 density). The interphase was washed and the pellet resuspended in 500 µl FACS-Buffer (PBS with calcium and magnesium containing 0.5% fetal calf serum and 0.5% NaN_3_). Samples were immediately analyzed^73^.

For the assessment of adhesion molecule expression, endothelial cells were cultured in 24-well plates. At 80–90% confluence, cells were treated with CM or serum free DMEM for different periods of time (4 or 24 h). After incubation, endothelial cells were harvested using Accutase (300 µl/well), collected by centrifugation and stained for 30 min at 4 °C with the following antibodies 1:20 in 100 µl FACS-Buffer: 1) PE conjugated anti-human CD54 (anti-ICAM-1); 2) PE conjugated anti-human CD62E (anti-E-selectin); 3) PE conjugated anti-human CD31 (anti-PECAM). Isotype-matched antibodies served as negative controls. After washing, cells were resuspended in 500 µl FACS-Buffer and analyzed.

For the assessment of circulating progenitor cells, 100 µl EDTA-whole blood from the tail vein was incubated for 30 min at 4 °C with the following antibody combination: polyclonal goat anti-mouse Sca-1/Ly6 antibody (1:12, R&D Systems) and polyclonal rabbit anti-cKit antibody (1:25, Bioss). After washing with PBS, samples were incubated for 30 min at 4 °C with the secondary antibodies Alexa Fluor 647 donkey anti-goat and Alexa Fluor 488 sheep anti-rabbit (each 1:500, life technologies), respectively. After washing, erythrocytes were lysed. After centrifugation, samples were resuspended in 500 µl FACS-Buffer and analyzed. Isotype-matched antibodies served as negative control. Gates were set at forward scatter (FSC) and sideward scatter (SSC), including lymphocytes and excluding monocytes and granulocytes^71^.

All samples were analyzed using the FACSCalibur flow cytometer (BD) with the Cell Quest Plus software.

### Statistical analysis

All data are presented as mean ± SEM. Groups were compared to Nx + vehicle by using one-way ANOVA along with post-hoc Dunnet’s test. Comparison among groups was performed by one-way ANOVA along with post-hoc Tukey’s test as indicated in Figure legends. For variables with skewed distribution according to the Kolmogorov-Smirnov test (e.g. aldosterone levels), statistics were based on the log-transformed data. Student’s t-test was used when appropriate. P < 0.05 was considered statistically significant. All analyses were performed using GraphPad Prism version 5.02 for windows.

## Electronic supplementary material

Supplemental Data
