# Supplementary material for: Bone marrow-derived cells and their conditioned medium induce microvascular repair in uremic rats by stimulation of endogenous repair mechanisms
Source: Sci Rep. 2017 Aug 25;7:9444. doi: 10.1038/s41598-017-09883-x (PMC5572734; doi:10.1038/s41598-017-09883-x)
Supplement: Supplementary file 1 — Supplemental Data [file 41598_2017_9883_MOESM1_ESM.doc]

**Bone marrow-derived cells and their conditioned medium induce microvascular repair in uremic rats by stimulation of endogenous repair mechanisms**

Lina Golle^1^, Hans U. Gerth^1^, Katrin Beul^1^, Barbara Heitplatz^2^, Peter Barth^2^, Manfred Fobker^3^, Hermann Pavenstädt^1^, Giovana S. Di Marco^1#^* & Marcus Brand^1#^.

^1^Department of Internal Medicine D, University Hospital Muenster, Muenster, Germany. ^2^Department of Pathology, University Hospital Muenster, Muenster, Germany. ^3^Centre for Laboratory Medicine, University of Münster, Münster

^#^These authors contributed equally to this work.

Correspondence and requests for materials should be addressed to G.S.D.M. (email: [giodimarco@gmail.com](mailto:giodimarco@gmail.com) or dimarco@uni-muenster.de)

**
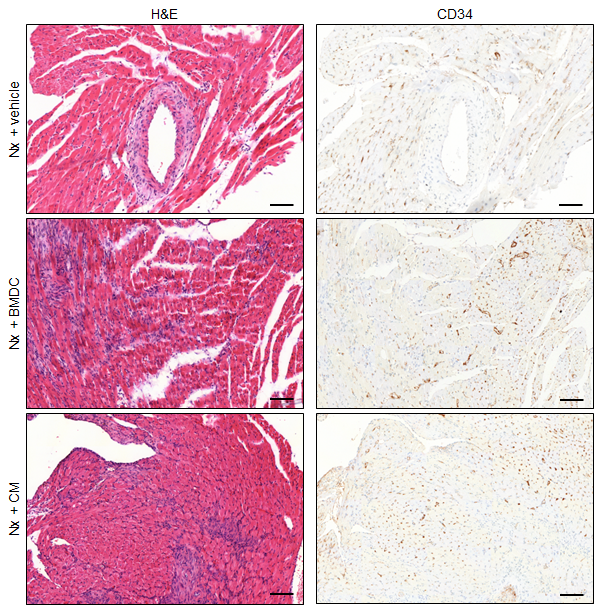
**

**Supplemental Figure1:** Histological and immunohistochemical analyses. Hematoxylin-eosin (H&E) staining evidences cell infiltrates in the hearts 14 days after 5/6 nephrectomy (Nx)/treatment. Immunohistochemical staining for CD34, a marker of vascular endothelial progenitor cells, shows positive cells close to but not within these infiltrates. Bar: 100 µm. BMDCs: bone marrow-derived cells (30 x 10^6^ per week); CM: BMDC-conditioned medium (1 mg protein per week).

**Supplemental Figure 2:** von Kossa staining. Histological analysis after von Kossa staining shows no evidence of mineralization in the hearts of rats 14 days after 5/6 nephrectomy (Nx) surgery and treatment with vehicle (NaCl), bone marrow-derived cells (BMDC, 30 x 10^6^ cells per week) or BMDC-conditioned medium (CM, 1 mg protein per week) in Sprague-Dawley rats. Tissues were treated with silver nitrate solution that reacts with calcium compounds within the tissue sample to form silver deposits. Calcified foci would be seen as black dots. Magnification, x20; scale bar: 50 µm. Arrows indicate interstitial cell infiltrate.

**Expanded Methods**

**Animal model**

Experiments were approved by a governmental committee on animal welfare and were performed in accordance with national animal protection guidelines.

For the animal model of CKD, renal injury was induced in 260 - 300 g, healthy male Sprague-Dawley rats (Charles River, Erkrath, Germany) by 5/6 nephrectomy (Nx) as described before.^1^ The surgery involved a midline incision, the ligation of 2-3 branches of the left renal artery and removal of the right kidney. The nephrectomy was carried out under 2-4% isoflurane. After surgery, rats were randomized into different groups: Nx + vehicle rats received saline injection (i.p.) once a week; Nx + BMDC rats received 30 x 10^6^ BMDCs/week (i.v.), isolated from 4-week old, healthy donor rats; Nx + CM rats received 1 mg total protein of CM per week. Sham operation consisted of midline incision and decapsulation of the right kidney. Rats were kept in pairs for 14 days with *ad libitum* access to water and food. They were fed standard diet containing 0.6% phosphorus and 0.6% calcium (Altromin maintenance diet 1324, Lage, Germany). On day 13, they were individually housed in metabolic cages for 24h for urine collection. Water intake, urine output and body weight were determined. Animals were then sacrificed, blood (serum) was collected by decapitation on isoflurane anesthesia, and hearts were excised, weighed and prepared for molecular and histological analyses as described below. Urine and blood samples were analyzed for electrolytes, protein (Bradford Blue; BioRad Laboratories, Arnsberg, Germany), creatinine (enzymatic assay; Creatinine-Pap, Roche Diagnostics, Mannheim, Germany), and blood urea nitrogen (urease-GLDH method) on a Roche Diagnostic analyzer (Modular P; Roche Diagnostics, Mannheim, Germany). Serum inorganic phoshate and calcium were measured according to the standard procedure recommended by the manufacturer with an automated Cobas 8000 analyzer system (Roche Diagnostics GmbH, Mannheim, Germany). All analyses were performed at the Centre for Laboratory Medicine at the University Hospital Münster (UKM).

To analyze engraftment of BMDCs, Nx was performed in 260-300 g male Lewis rats as described above. Rats were randomized into two groups: rats received either vehicle (i.p. saline injection once per week) or 30 x 10^6^ eGFP^+^ BMDCs (i.v. injection once per week). BMDCs were isolated from 4-week old, GFP-transgenic Lewis rats. After 14 days, hearts were excised und submitted to flow cytometry analysis as described below.

**BMDC isolation and BMDC-conditioned medium**

For the isolation of BMDCs, tibia, femur and humerus were removed from 4-week old, healthy Sprague-Dawley or GFP-transgenic Lewis rats and placed in ice-cold PBS with 10 U/ml heparin as modified from Yang et al.^2^ The whole bone marrow was flushed out with PBS and filtered using a 70 µm Cell Strainer (Falcon, Durham, NC, USA). The suspension was centrifuged at 500 *x g* for 20 min without brake. To remove erythrocytes, the cells were incubated in 1 x lysing solution (Beckham Coulter, Marseille, France) for 10 min, followed by another centrifugation at 500 *x g* for 10 min without brake. The cell pellet was resuspended in 10 ml PBS. After cell counting and centrifugation at 500 *x g* for 10 min, cells were resuspended in serum free DMEM containing 2 mM L-glutamine, and 50 U/ml each of penicillin/streptomycin at the concentration needed for experiments or stored in liquid nitrogen using 50% fetal calf serum (PAA Laboratories, Pasching, Austria), 40% DMEM (Biochrom, Berlin, Germany) and 10% DMSO.

For BMDC conditioned medium (CM), isolated BMDCs were seeded either on a 24-well plate at a concentration of 2 x 10^6^ cells/well (for *in vitro* assays) or in a cell culture flask at a concentration of 60 x 10^6^ cells/5 ml culture medium (for *in vivo* experiments). Cells were incubated with serum free for 24 h at 37°C and 5% CO_2_, before they were centrifuged at 1000 *x g* for 6 min. The supernatant was transferred to a fresh Eppendorf cup and stored at -20°C. For the treatment of rats, the CM was concentrated using Amicon Ultra-4 Centrifugal Filters (3K) (Merck, Darmstadt, Germany) to a final concentration of 2 mg/ml total protein as modified from van Koppen et al.^3^

**Capillary density**

For the staining of capillaries in the heart, Isolectin B4, a marker of endothelial cells, was used as previously described.^4^ After deparaffinization and rehydration, three µm thick organ sections of 4% PFA-fixed hearts were incubated with 3% hydrogen peroxide in PBS for 15 min, washed with PBS, blocked with 0.3 mg/ml bovine serum albumin (BSA) in PBS for 30 min and incubated with biotinylated Isolectin B4 (Vector, Burlingame, CA, USA) 1:50 in PBS + 0.1 mg/ml BSA overnight at 4°C. For detection, the avidin-biotin complex (ABC Kit; Vector, Burlingame, CA, USA) was used, before incubation with diaminobenzidine (DAB) solution (Dako, Carpinteria, CA, USA) following manufacturer’s instructions. For visualization of cell borders and cell nuclei, a hematoxylin-eosin (H&E) staining was performed.

Digitized pictures (8 separate high-power fields per section) were taken from the left ventricle by using a Carl Zeiss microscope and the AxioVisonLE Release 4.7.1 software with a 100x magnification. Heart vascularization was determined by counting the number of blood vessels per cardiomyocyte.^4^

**Histological Analysis and assessment of fibrosis**

To determine the extent of fibrosis, the collagen in paraffin-embedded tissue sections (three µm thick) was stained with Picrosirius red, as previously described.^5^ In brief, after deparaffinization and rehydration, heart sections were incubated with hematoxylin (Weigert’s iron hematoxylin kit; Merck, Darmstadt, Germany) for 8 min, washed with tab water and incubated with Sirius Red Solution (picric acid; Waldeck, Münster, Germany) for 1 h, and sections were washed twice with acidified water (0.5% glacial acetic acid). Digitized pictures (8 separate high-power fields per section) were taken from the left ventricle by using a Carl Zeiss microscope and the AxioVisonLE Release 4.7.1 software with a 20x magnification. Extent of fibrosis was calculated using ImageJ software.

Kidney and heart histology by H&E (Roth, Karslruhe, Germany) and von Kossa stainings (Merck, Darmstadt, Germany) were performed according to manufacturers’ instructions.

**Immunohistochemistry**

Immunohistochemistry was performed on three µm thick histological sections of 4% PFA-fixed paraffin-embedded tissue samples using the Ventana OptiView IHC Detection Kit following standardized protocols of the manufacturer. The following primary antibodies were used: monoclonal α-smooth muscle actin antibody (αSMA; Cell Marque, clone 1A4) at a ready to use dilution of 0.02 µg/ml; monoclonal CD163 (Cell Marque, clone MRQ-26) at a ready to use dilution of 0.17 µg/ml; and monoclonal CD4 (Ventana, clone SP 35) at a ready to use dilution of 2.5 µg/ml. Slides were digitized by an automated digital slide scanner (Mirax Midi; 3DHISTECH Ltd, Budapest, Hungary) and pictures were analyzed using the Pannoramic Viewer Software (3DHISTECH Ltd, Budapest, Hungary).

**Gene and miRNA expression**

Total RNA was isolated from heart tissue stored in RNAlater (Qiagen, Hilden, Germany) or EA.hy926 cells directly harvested in RLT-Buffer (RNEasy Mini Kit; Quiagen, Hilden Germany).

The gene expression was then analyzed by real-time PCR using the SYBR Select Master Mix (Applied Biosystems, Darmstadt, Germany) as described before.^5^ The relative gene expression was analyzed using the 2^-ΔΔCt^ method and 18S as reference gene. Results were log-transformed before statistical analysis. Rat primer sequences are: MCP-1 forward 5´-gctgctactcattcactggcaa-3´ and reverse 5´-tgctgctggtgattctcttgta-3´; ICAM forward 5´-cgggagatgaatggtacc-3´ and reverse 5´-gcggtaataggtgtaaatgg-3´; Il-6 forward 5´-ttggatggtcttggtccttagcc-3´ and reverse 5´-tcctaccccaacttccaatgctc-3´; Il-10 forward 5´-ctcccctgtgagaataaaagcaag-3´ and reverse 5´-agtgtcacgtaggcttctatgc-3´; 18S forward 5´-gcggcttaatttgactcaacac-3´ and reverse 5´-agacaaatcgctccaccaacta-3´. Human primer sequences are: MCP-1 forward 5´-tgcagaggctcgcgagcta-3´ and reverse 5´-caggtggtccatggaatcctga-3´; ICAM forward 5´-tgtgaccagcccaagttgtt-3´ and reverse 5´-agtccagtacacggtgagga-3´; IL-6 forward 5´-acatcctcgacggcatctca-3´ and reverse 5´-caccaggcaagtctcctcatt-3´; 18S forward 5´-ctcaacacgggaaacctcac-3´ and reverse 5´-cgctccaccaactaagaacg-3´.

For analysis of miRNA expression, the following commercial kits were used: mirVana miRNA isolation Kit (Invitrogen), [TaqMan Advanced miRNA cDNA Synthesis Kit](https://www.thermofisher.com/order/catalog/product/A28007?ICID=search-product) (Applied Biosystems) and the TaqMan Advanced miRNA Assays: rno-miR-126-3p, rno-miR-126-5p, rno-miR-222-3p and rno-let-7g-5p. The relative expression was analyzed using the 2^-ΔΔCt^ method and rno-let-7g-5p as endogenous control. Results were log-transformed before statistical analysis.

All measurements were performed at the Integrated Functional Genomics facilities (IFG, Münster, Germany) using the ABI PRISM 7900 Sequence Detection System (Applied Biosystems, Darmstadt, Germany).

**Cytokine Array**

The cytokine array was performed using the Rat Cytokine Antibody Array C2 kit (RayBio, Norcross, GA, USA) following the manufacturer’s instructions. In short, membranes were incubated overnight with concentrated CM at 4°C under gentle agitation. Chemiluminescent signals were detected using the FluorChem FC2 Imaging System (Alpha Innotech, Miami, FL, USA).

**ELISA**

Commercial ELISA Kits were used to determine levels of stem cell factor (mouse SCF; R&D Systems), stromal cell derived factor 1 (rat SDF-1; Cloud Clone Corp.), granulocyte-colony stimulating factor (rat G-CSF; CUSABIO) and parathyroid hormone (rat intact PTH; Immutopics) in serum and/or CM. Rat serum aldosterone was determined by chemiluminescent immunoassay technology with an automated LIAISON® analyzer system (DiaSorin Deutschland GmbH, Dietzenbach, Germany).

**Endothelial cell culture**

EA.hy926 cells, a human umbilical vein endothelial cell line that expresses highly differentiated functional characteristic of human vascular endothelium,^6, 7^ were grown in DMEM containing 5% fetal calf serum (PAA Laboratories, Pasching, Austria), 2 mM L-glutamine, and 50 U/ml each of penicillin/streptomycin at 37°C in an atmosphere of 5% CO_2_ in air.

For gene expression, cells were cultured in 24-well plate. At 80-90% confluence, cells were treated with CM or serum free DMEM for different periods of time (4 or 24 h). After incubation, endothelial cells were harvested with lysis buffer (RNEasy Mini Kit; Quiagen, Hilden Germany) and submitted to RNA extraction and gene analysis as described above. Additional culture conditions and treatments are described below.

**Viability assay**

The effects of CM on endothelial cell viability was assessed using MTT assay.^8^ In brief, endothelial cells were cultured in a 96-well plate (80-90% confluence, 100 µl medium/well) were treated with CM or serum free DMEM for 24 h. At the end of the incubation time, 5 µl of MTT-solution (5 mg/ml in NaCl 0.9%; Sigma-Aldrich, St. Louis, MO, USA) were added to each well, and cells were further incubated for 3 h. Medium was then removed and cells were solubilized with a lysing solution (100 µl/well; 100 ml 20% SDS, 34 ml N_3_N-dimethylformamide, 16 ml destilled water) overnight. The absorbance was measured at 590 nm in a microplate reader (Tecan, Salzburg, Austria).

**Tube formation assay**

In order to analyze the proangiogenic activity of CM, a tube-formation assay was performed using matrigel.^9^ A µ-slide Angiogenesis (ibidi, Martinsried, Germany) was coated with 10 µl matrigel/well (corning, Bedford, MA, USA) and incubated for 5 h at 37°C. EA.hy926 cells were seeded on the matrigel at a concentration of 1 x 10^4^/well with either CM or serum free DMEM as control and incubated for 17 h. Pictures were taken with a 4x magnification and analyzed using the Angiogenesis Analyzer for ImageJ (Gilles Carpentier). Total tube length in pixel was compared.

**Adhesion Assay**

Leukocyte- and BMDC-endothelial adhesion was determined as previously described with some minor modifications.^10^ In brief, peripheral blood leukocytes were separated from EDTA-blood of healthy volunteers by density gradient centrifugation (Lymphocyte Separation Medium, 1077 density, PAA Laboratories, Pasching, Austria). BMDC were isolated as described above. The cells were labeled with calcein-AM (3 μM; eBioscience, San Diego, CA, USA) in phenol red-free RPMI containing 5% fetal calf serum (Washing medium) for 30 min at 37°C protected from light. Cells were washed twice to remove excess calcein-AM and resuspended in binding medium (phenol red-free RPMI containing 2% fetal calf serum). The cells were then counted and added (150-300 x 10^3^/well, 100 µl volume) to confluent monolayers of EA.hy926 cells that had been grown in 96-well plates and treated for 4 hours with serum free DMEM or CM. The amount of labeled cells added was assessed by measuring the fluorescence signal (total signal) using a fluorescence spectrometer equipped with a microplate reader (Ex: 485 nm, Em: 530 nm; Tecan, Salzburg, Austria). After 60 or 180 min incubation at 37°C for leukocytes and BMDCs, respectively, non-adherent cells were removed by washing 2-3 times with pre-warmed washing medium. The fluorescent signal was reassessed by the microplate reader (adherent signal) in the presence of 100 µl binding medium. The percentage of leukocytes adhering to the endothelial monolayer was calculated by the formula: % adherence = (adherent signal/total signal) x 100.

**Flow cytometry**

For the analysis of BMDC-engraftment, heart and kidney were mechanically shredded using a scalpel and pressed through a 70 µm Cell Strainer (Falcon, Durham, NC, USA). After washing with PBS, the suspension was processed by density gradient centrifugation (Lymphocyte Separation Medium, 1077 density, PAA Laboratories, Pasching, Austria) and centrifuged at 400 *x g* for 30 min without brake. The interphase was transferred to a fresh Falcon tube and centrifuged at 300 *x g* for 5 min. The supernatant was discarded and the pellet resuspended in 500 µl FACS-Buffer (PBS with calcium and magnesium containing 0.5% fetal calf serum and 0.5% NaN_3_). Samples were immediately analyzed.^11^

For the assessment of adhesion molecule expression, endothelial cells were cultured in 24-well plates. At 80-90% confluence, cells were treated with CM or serum free DMEM for different periods of time (4 or 24 h). After incubation, endothelial cells were harvested using Accutase (300 µl/well; Sigma-Aldrich, St. Louis, MO, USA), collected by centrifugation and stained for 30 min at 4°C with the following antibodies 1:20 in 100 µl FACS-Buffer: 1) PE conjugated anti-human CD54 (anti-ICAM-1, BD Biosciences, San Jose, CA, USA); 2) PE conjugated anti-human CD62E (anti-E-selectin; eBioscience, San Diego, CA, USA); 3) PE conjugated anti-human CD31 (anti-PECAM; BD Biosciences, San Jose, CA, USA). Isotype-matched antibodies served as negative controls. After washing with PBS, cells were resuspended in 500 µl FACS-Buffer and analyzed. Twenty thousand events were analyzed in the green (FITC) channel.

For the assessment of circulating progenitor cells, 100 µl EDTA-whole blood from the tail vein was incubated for 30 min at 4°C with the following antibody combination: polyclonal goat anti-mouse Sca-1/Ly6 antibody (1:12, R&D Systems, Minneapolis, MN, USA) and polyclonal rabbit anti-cKit antibody (1:25, Bioss, Woburn, MN, USA). After washing with PBS, samples were incubated for 30 min at 4°C with the secondary antibodies Alexa Fluor 647 donkey anti-goat and Alexa Fluor 488 sheep anti-rabbit (each 1:500, life technologies, Eugene, OR, USA), respectively. After washing, samples were incubated with 1x lysing solution (Beckham Coulter, Marseille, France) for the lysis of erythrocytes for 10 min. After centrifugation, samples were resuspended in 500 µl FACS-Buffer and analyzed. Isotype-matched antibodies served as negative control. Gates were set at forward scatter (FSC) and sideward scatter (SSC), including lymphocytes and excluding monocytes and granulocytes.^9^

All samples were analyzed using the FACSCalibur flow cytometer (BD, Franklin Lakes, NJ, USA) with the Cell Quest Plus software (BD, Franklin Lakes, NJ, USA).

**Statistical analysis**

All data are presented as mean ± SEM. Groups were compared to Nx + vehicle by using one-way ANOVA along with post-hoc Dunnet’s test. Comparison among groups was performed by one-way ANOVA along with post-hoc Tukey’s test as indicated in Figure legends. For variables with skewed distribution according to the Kolmogorov-Smirnov test (e.g. aldosterone levels), statistics were based on the log-transformed data. Student’s t-test was used when appropriate. P<0.05 was considered statistically significant. All analyses were performed using GraphPad Prism version 5.02 for windows.

References

1. Di Marco, G. S. *et al.* Soluble Flt-1 links microvascular disease with heart failure in CKD. *Basic Res. Cardiol.* **110**, 30 (2015).

2. Yang, J. D. *et al.* The isolation and cultivation of bone marrow stem cells and evaluation of differences for neural-like cells differentiation under the induction with neurotrophic factors. *Cytotechnology* **66**, 1007-1019 (2014).

3. van Koppen, A. *et al.* Human embryonic mesenchymal stem cell-derived conditioned medium rescues kidney function in rats with established chronic kidney disease. *PLoS. One.* **7**, e38746 (2012).

4. Di Marco, G. S. *et al.* Cardioprotective effect of calcineurin inhibition in an animal model of renal disease. *Eur. Heart J.* **32**, 1935-1945 (2011).

5. Di Marco, G. S. *et al.* Treatment of established left ventricular hypertrophy with fibroblast growth factor receptor blockade in an animal model of CKD. *Nephrol. Dial. Transplant.* **29**, 2028-2035 (2014).

6. Edgell, C. J., McDonald, C. C. & Graham, J. B. Permanent cell line expressing human factor VIII-related antigen established by hybridization. *Proc. Natl. Acad. Sci. U. S. A* **80**, 3734-3737 (1983).

7. Edgell, C. J. *et al.* Endothelium specific Weibel-Palade bodies in a continuous human cell line, EA.hy926. *In Vitro Cell Dev. Biol.* **26**, 1167-1172 (1990).

8. Denizot, F. & Lang, R. Rapid colorimetric assay for cell growth and survival. Modifications to the tetrazolium dye procedure giving improved sensitivity and reliability. *J. Immunol. Methods* **89**, 271-277 (1986).

9. Di Marco, G. S. *et al.* High phosphate directly affects endothelial function by downregulating annexin II. *Kidney Int.* **83**, 213-222 (2013).

10. Kim, I., Moon, S. O., Park, S. K., Chae, S. W. & Koh, G. Y. Angiopoietin-1 reduces VEGF-stimulated leukocyte adhesion to endothelial cells by reducing ICAM-1, VCAM-1, and E-selectin expression. *Circ. Res.* **89**, 477-479 (2001).

11. Oliveira-Sales, E. B. *et al.* Mesenchymal stem cells (MSC) prevented the progression of renovascular hypertension, improved renal function and architecture. *PLoS. One.* **8**, e78464 (2013).
